# Multi-attention Mechanism for Enhanced Pseudo-3D Prostate Zonal Segmentation

**DOI:** 10.1007/s10278-025-01401-0

**Published:** 2025-02-28

**Authors:** Chetana Krishnan, Ezinwanne Onuoha, Alex Hung, Kyung Hyun Sung, Harrison Kim

**Affiliations:** 1 Department of Biomedical Engineering, The University of Alabama at Birmingham, Birmingham, AL 35294 USA; 2 Department of Radiology, The University of California Los Angeles, Los Angeles, CA 90404 USA; 3 Department of Radiology, The University of Alabama at Birmingham, 1720 2Nd Avenue South, VH G082, Birmingham, AL 35294 USA

**Keywords:** Prostate MRI image segmentation, Deep learning, Attention variant, Feature map, Dot product

## Abstract

This study presents a novel pseudo-3D Global–Local Channel Spatial Attention (GLCSA) mechanism designed to enhance prostate zonal segmentation in high-resolution T2-weighted MRI images. GLCSA captures complex, multi-dimensional features while maintaining computational efficiency by integrating global and local attention in channel and spatial domains, complemented by a slice interaction module simulating 3D processing. Applied across various U-Net architectures, GLCSA was evaluated on two datasets: a proprietary set of 44 patients and the public ProstateX dataset of 204 patients. Performance, measured using the Dice Similarity Coefficient (DSC) and Mean Surface Distance (MSD) metrics, demonstrated significant improvements in segmentation accuracy for both the transition zone (TZ) and peripheral zone (PZ), with minimal parameter increase (1.27%). GLCSA achieved DSC increases of 0.74% and 11.75% for TZ and PZ, respectively, in the proprietary dataset. In the ProstateX dataset, improvements were even more pronounced, with DSC increases of 7.34% for TZ and 24.80% for PZ. Comparative analysis showed GLCSA-UNet performing competitively against other 2D, 2.5D, and 3D models, with DSC values of 0.85 (TZ) and 0.65 (PZ) on the proprietary dataset and 0.80 (TZ) and 0.76 (PZ) on the ProstateX dataset. Similarly, MSD values were 1.14 (TZ) and 1.21 (PZ) on the proprietary dataset and 1.48 (TZ) and 0.98 (PZ) on the ProstateX dataset. Ablation studies highlighted the effectiveness of combining channel and spatial attention and the advantages of global embedding over patch-based methods. In conclusion, GLCSA offers a robust balance between the detailed feature capture of 3D models and the efficiency of 2D models, presenting a promising tool for improving prostate MRI image segmentation.

## Introduction

Semantic-level image segmentation is fundamental to visual representation learning. It is closely linked to challenges in clinical visual recognition and machine learning, such as metric, few-shot, and unsupervised learning [1]. Transformers were introduced initially for natural language processing (NLP) [2] and have recently emerged as the leading foundation model in computer vision, mainly due to the groundbreaking work of the Vision Transformer (ViT) [3]. Their success has since extended across multiple vision tasks like image classification, recognition, and segmentation. Research on semantic-level image segmentation has primarily concentrated on feature learning using convolutional neural networks (CNNs), with some studies also exploring re-ranking techniques, such as graph-based methods [4]. Feature learning techniques can be classified into two main categories: local descriptors, similar to SIFT [5], where an image is represented by several hundred vectors, and global descriptors, where a single vector represents an image.

Deep learning has notably advanced the performance of global descriptors, enabling more efficient searches. Research on global learning has predominantly focused on spatial attention [6]. The need for compact and discriminative representations that can effectively handle visual detection has naturally led to the development of spatial attention methods [7]. Various forms of attention mechanisms have been investigated across different domains of computer vision. These include channel attention [8]; local attention applied independently to features within a representation [9]; global attention, which depends on the relations between elements [10]; and integrations of these methods [11]. However, most studies have been confined to exploring only single or double integrations of attention, with attention mechanisms not always effective and applications varying across different dependencies [12–14].

Prostate cancer (PCa) ranks among the most common cancers and is a leading cause of cancer-related mortality in men in the United States [15]. Multi-parametric MRI (mpMRI), which includes T2-weighted imaging, diffusion-weighted imaging, and dynamic contrast-enhanced MRI, has emerged as the preferred non-invasive approach for prostate cancer diagnosis [16]. The Prostate Imaging Reporting and Data System (PI-RADS) [17], the current clinical framework for analyzing mpMRI results, suggests that lesions of concern should be assessed differently in distinct prostate regions, such as the transition zone (TZ) and peripheral zone (PZ), due to variations in image features and the prevalence of cancer. Furthermore, the size of the TZ is often utilized in clinical settings for evaluating and tracking benign prostate hyperplasia (BPH) [18].

However, manually annotating prostate zones is often time-consuming, and the variability may occur across the annotator’s experience. Thus, there is a critical need for reliable and robust automated zonal segmentation methods. 3D convolutional neural networks (CNNs) significantly improve prostate zone segmentation in MRI by leveraging spatial context across imaging planes, leading to more precise and consistent results compared to 2D methods [19]. However, these networks are computationally demanding, requiring substantial memory and processing power, which can limit their feasibility in clinical settings [20]. The high resource requirements make scaling to larger datasets or higher-resolution images challenging, necessitating a balance between accuracy and efficiency in their practical application [21].

Previous studies have demonstrated attention–transformer semantic segmentation tasks for medical object detection. Ling et al. introduced DANet for pixel-wise dense labeling, incorporating both channel and spatial attention blocks into the output path of a dilated Fully Convolutional Network (FCN) to capture semantic relations within various dimensions in the input image [22]. Mou et al. employed a novel integration strategy in recurrent and convolutional models for structure segmentation and curve recognition, combining channel and spatial attention by summing their outputs [23]. Lyu et al. employed the swift concatenation-sequential method to merge the results of the dual attention modules [24]. An alternative dual attention method was introduced, where the attention modules are applied in sequence [25]. Shen et al. demonstrated that a single attention type can capture the global and local dependencies [26]. To solve the 3D network limitations, CAT-Net introduced a novel cross-slice attention mechanism within a transformer framework to capture and utilize cross-slice information effectively, improving segmentation accuracy and consistency, particularly in the peripheral zones of the prostate [27]. An early example of global attention in image classification is the non-local neural network (NLNet) [28], which introduced global spatial attention by enabling interactions between any two spatial points. Later studies further explored similar global spatial attention techniques [29, 30]. Additionally, advancements in global channel attention mechanisms have allowed interactions between different channels [31, 32]. However, global attention has typically been applied separately to spatial or channel interactions, rarely addressing both simultaneously.

This study analyzes different attention mechanisms and applies them to semantic image segmentation, focusing on how they work together in a pseudo-3D setting. We introduce GLCSA, a network that combines global and local attention with inter-slice interactions for better segmentation, providing a 3D-like accuracy and 2D-like computational speed. Using spatial and channel attention, our method improves prostate zonal segmentation while keeping the system efficient. We propose a simpler patch embedding technique using 2D average pooling and depth-wise convolutions to reduce complexity. This approach boosts segmentation accuracy, especially in PZ regions, while keeping computational demands low, as demonstrated through tests on U-Net models and two prostate MRI datasets.

## Materials and Methods

### Proposed Architecture

**Fig. 1:**
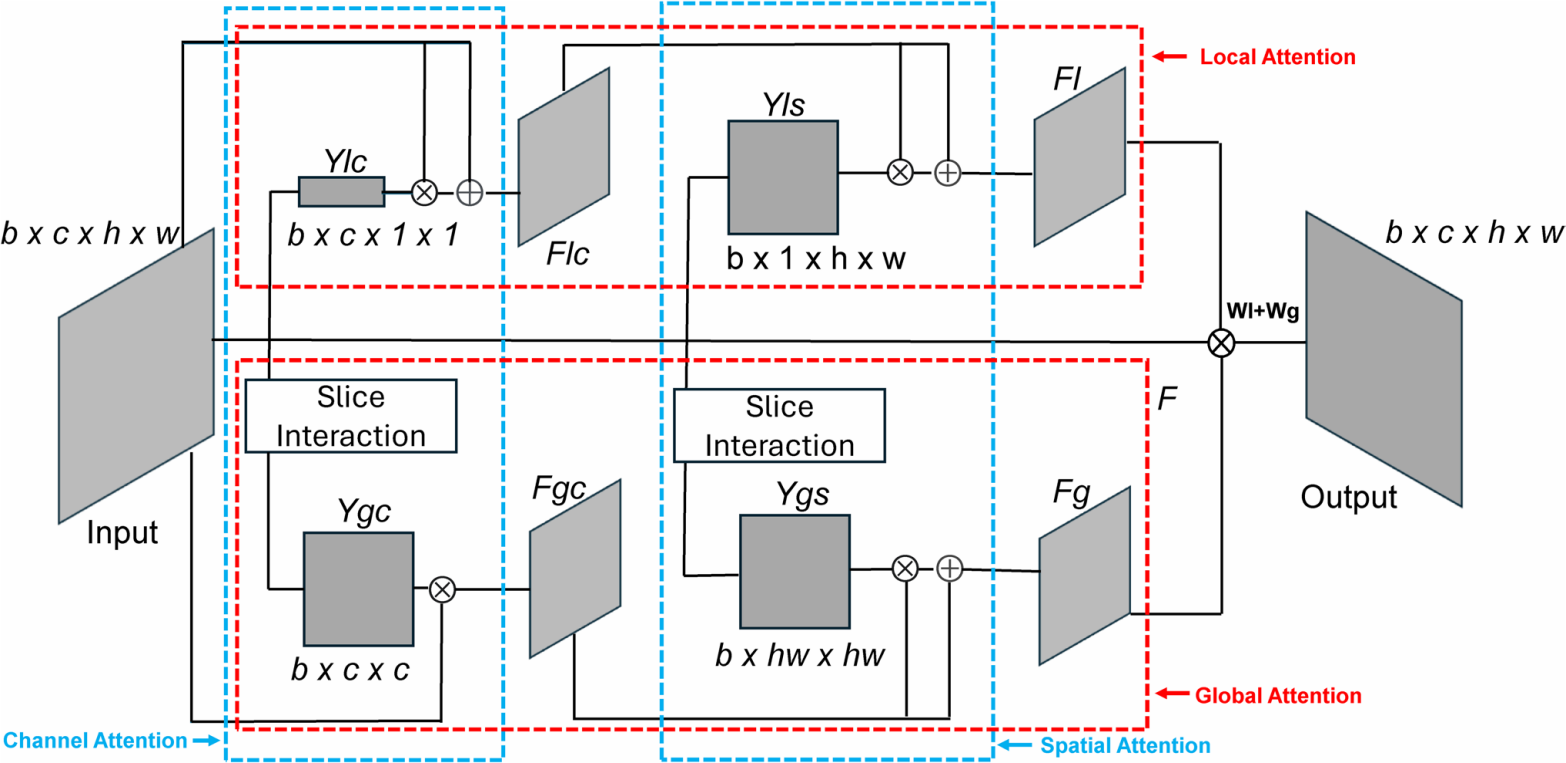
Schematic of the GLCSA module. GLCSA uses local attention, where channels and locations are weighted based on context and global attention relies on pairwise interactions. Four attention maps are generated—local spatial (*Yls*), local channel (*Ylc*), global spatial (*ygs*), and global channel (*Ygc*). These maps weight the input feature map *F* into global (*Fg*) and local (*Fl*) attention maps, which are merged with *F* to produce the final feature map (*Fgl*). A slice interaction mechanism also facilitates interactions across input slices, leveraging the batch dimension as depth for pseudo-3D processing

Figure 1 shows the architecture of the GLCSA module, which outlines the key components: a feature tensor *F* with dimensions *b* × *c* × *h* × *w*, where *c* is the number of channels and *b* × *h* × *w* indicates the spatial resolution. The global attention mechanism first facilitates interactions across channels, producing a *b* × *hw* × *hw* spatial map (*Ygs*) and *b* × *c* × *c* channel map (*Ygc*). In parallel, the local attention mechanism gathers contextual details from the image, generating a channel map (*Ylc*) and a spatial map (*Yls*) of shapes *b* × *c* × *1* × *1* and* b* × *1* × *h* × *w* respectively. These output maps are then merged with the original feature tensor through a fusion process to create the final feature map (*F*), which is then pooled spatially into a global descriptor. An additional slice interaction mechanism is introduced where the feature tensor *F* is processed along the batch dimension (*b*), treating the batch as the pseudo-depth layer. This technique aids in identifying relationships between various samples within the batch, enhancing the contextual understanding along the depth dimension. The weights from local (*Wl*) and global (*Wg*) perspectives will then be fused to get the final attention map (*F*).

**Fig. 2:**
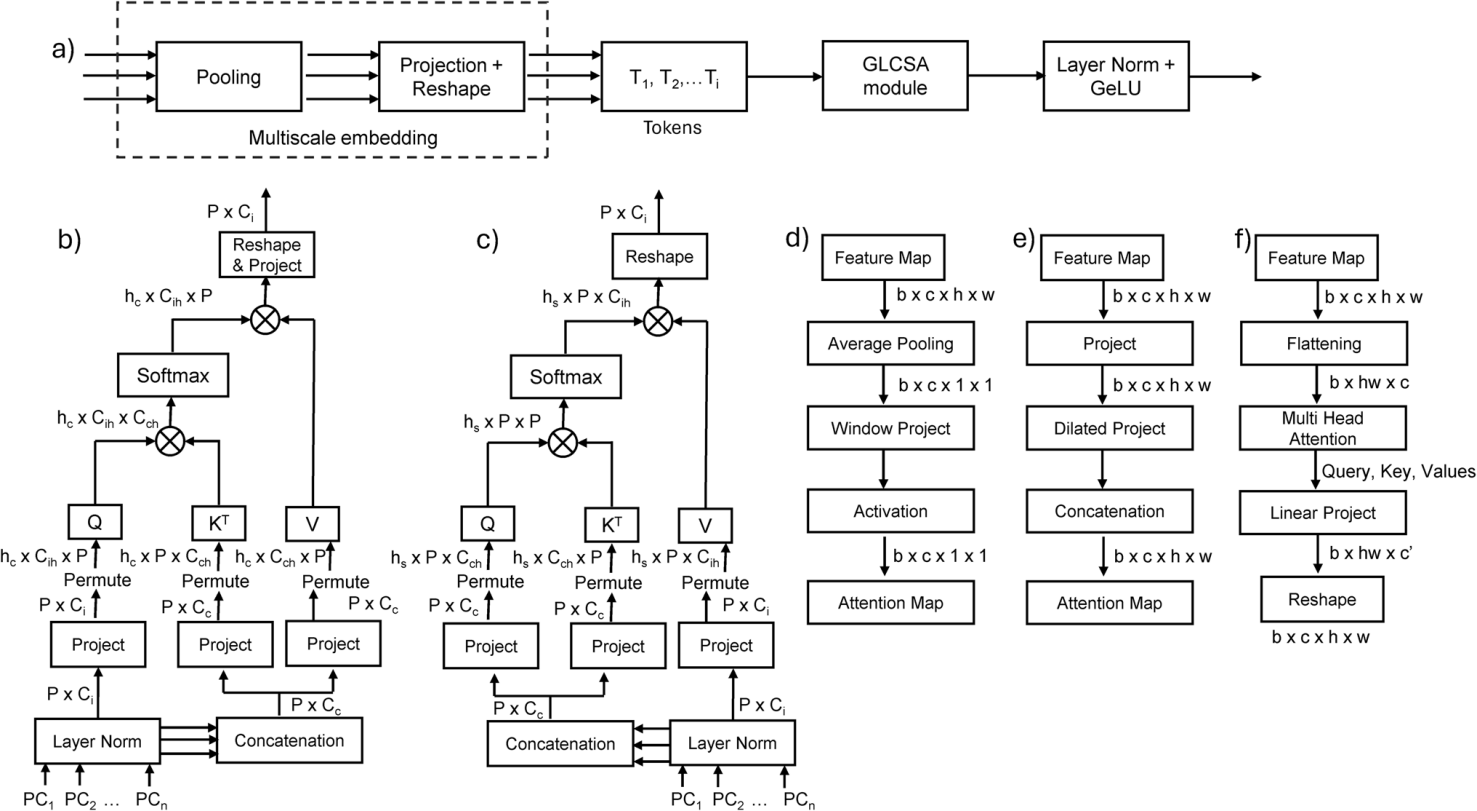
Schematics of GLCSA block and submodules. **a** Schematics of GLCSA block containing GLCSA module. **b**–**f** Schematics of GLCSA submodules such as **b** global channel attention (GCA), **c** global spatial attention (GSA), **d** local channel attention (LCA), **e** local spatial attention (LSA), and **f** slice interaction module (SIM)

Figure 2a illustrates the integration of the suggested GLCSA module into the GLCSA block. The structure of the GLCSA module can be adjusted to accommodate any number of encoder layers. For a network with *n* + 1 encoder layers, which are scaled differently, the GLCSA module processes the multiscale features from the first *n* layers, which is typically each stage’s convolutional layer outputs, producing improved representations that are then connected to the corresponding *n* decoder layers. As shown in Fig. 2a, the GLCSA block consists of two main phases. The first phase uses a multiscale embedding component that gathers encoder tokens. In the second phase, the GLCSA module captures extensive interdependencies between these tokens. This is followed by layer normalization and a GeLU activation before upsampling the tokens to link them to their respective decoder layers.

### Multiscale Embedding

We drove patches from *n* distinct stages of the encoder, corresponding to the skip-connection layers at various scales. Each of these encoder stages is represented as ${\text{EE}}_{i}\in {R}^{{C}_{i} x \frac{H}{{2}^{i-1}} x \frac{W}{{2}^{i-1}}}$ for *i* = 1, 2, …, *n* is associated with a specific patch size ${P}_{si} = \frac{{P}_{s}}{{2}^{i-1}}$. We extracted these patches by performing 2D mean pooling [33], where the pooling and stride parameters were determined by the corresponding patch size *P*_*si*_. Once the 2D patches were flattened, a 1 × 1 depth-wise convolution was applied to project the patches. The flattened patches *T*_*i*_, where ${\text{T}}_{i}\in {R}^{{C}_{i} x P}$ for the i-th encoder stage, are denoted as *T*_*i*_ = *DepthConv1D*(*Permute*(*AvgPool2D(EE*_i_))). Here, *P* represents the number of patches consistent across all *T*_i_ to enable the application of attention and slice interaction mechanisms on these tokens. This embedding process aimed to transform the multiscale feature maps obtained from different stages of an encoder into a uniform set of tokens or patches. These tokens served as a compact representation of the spatial features at each scale, making them compatible for subsequent processing.

### Global Channel Attention (GCA)

Figure 2b illustrates a global channel attention (GCA) module. The process starts with layer normalization (LN) [34] applied to each token *T*_*i*_. Instead of using conventional self-attention, where linear projections are employed, we drew inspiration from recent studies [35, 36] that have successfully integrated convolutions into self-attention. These studies highlighted how convolutions, particularly depth-wise convolutions, could enhance the ability to capture local features while lowering computational complexity [37, 38]. In our approach, after normalizing the tokens, the tokens *T*_*i*_ where *i* = 1 to *n* iterations were fused along the batch channel dimension to generate the values and keys, referred to as *T*_*c*_. The original tokens *T*_*i*_ were then used to query. We replaced the standard linear layers in the self-attention mechanism with depth-wise convolutional layers of size 1 × 1 to take advantage of the locality and efficiency offered by depth-wise convolutions. This adjustment allowed us to maintain the benefits of local information capture while streamlining the computational process.

To implement channel attention, we first transposed the matrices for keys (*K*), values (*V*), and queries (*Q*_*i*_). This transformation reformulated GCA as $GCA({Q}_{i}, K, V) = Activation(\frac{{Q}_{i}^{T}K}{\sqrt{{C}_{C}}}){V}^{T}$, where the scaling factor is represented by $\frac{1}{\sqrt{{C}_{C}}}$, determined based on the dimensionality of the query and key vectors. The activation function was softmax, which computed the similarity between the queries and keys and produced weights. These weights were applied to the values, resulting in a weighted sum output (Ygc). After the attention output was generated, it was processed through depth-wise convolutional projections. Finally, the resulting outputs were fed into the next module—local channel attention (LCA) module for further refinement and integration.

### Global Spatial Attention (GSA)

Figure 2c illustrates a global spatial attention (GSA) module. First, the normalized outputs *T*_*i*_ are concatenated along the channel dimension. The outputs are used as queries and keys, and each token *T*_*i*_ is employed as the value. To perform the projection, we apply 1 × 1 depth-wise convolutions. The GSA mechanism is formulated as $GSA({Q}_{i}, K, V) = Activation(\frac{Q{K}^{T}}{\sqrt{{d}_{k}}}){V}^{T}$. Here, the scaling factor is represented by $\frac{1}{\sqrt{{d}_{k}}}$, determined based on the dimensionality of the query and key vectors. For a multi-head configuration, the *d*_*k*_ is given by $\frac{{C}_{c}}{{H}_{c}}$, where *H*_*c*_ represents the number of attention heads and *C*_*c*_ represents the total number of channels*.*

### Local Channel Attention (LCA)

Figure 2d illustrates the LCA module. Like the ECA-Net approach [39], the LCA module begins by applying depth-wise convolutions to the input tokens *T*_*i*_ to generate local feature maps for queries, keys, and values. These convolutions focus on local patterns within smaller regions or patches of the input data. A sliding window approach separates the input tensor *F* into local patches [40]. This window slides across the input, extracting localized regions for separate processing. Following the principle of ECA-Net, after extracting local patches, the LCA module captures local channel attention by reducing each local feature map to a tensor of shape *b* × *c* × *1* × *1* through average pooling (AP) denoted as ${F}_{GAP}^{l}=AP({F}^{l})$, where *F*^*l*^ is the local patch of *F*. A 1D depth-wise convolution is then applied along the channel dimension using a kernel size *k*, which controls the extent of cross-channel interaction. This convolution enables the neural network to emphasize the most significant channels, enhancing the model’s sensitivity to important local features. The resulting tensor is passed through a sigmoid activation function to produce the *b* × *c* × *1* × *1* local channel attention map (*Ylc*), emphasizing the most informative channels in the local regions as follows: $Ylc = \sigma (Depth Conv1D({F}_{GAP}^{l}, k)$. The computed local attention map (*Ylc*) is then used to reweigh the local patches, emphasizing the channels that contain the most relevant information as follows: ${F}_{c}^{l}=F \times Ylc + F$. This reweighted tensor is reshaped and permuted for compatibility with the attention mechanism, where scaled dot-product attention is applied. This attention mechanism computes the similarity between the queries and keys within each local patch, determining the attention weights. It is then weighed with the GCA output to get the final Global–Local Channel Attention (GLCA) output (*Fl*) as follows: $Fl = {F}_{c}^{l} \times Ygc + {F}_{c}^{l}$.

### Local Spatial Attention (LSA)

Figure 2e shows the schematic of the local spatial attention (LSA) module, which captures and leverages local spatial features within the input data by employing convolutional operations and attention mechanisms. Initially, the process starts by applying a 1 × 1 convolutional layer to the input feature tensors (queries, keys, and values), which reduces the channel dimensions to a suitable size for subsequent attention operations. This reduction prepares the features for both global and local attention processing. The transformed query, key, and value tensors are reshaped and permuted to fit the multi-head attention format. This setup allows the multi-head attention mechanism to effectively capture local patterns by applying scaled dot-product attention. The scale factor, computed as $\frac{1}{\sqrt{{d}_{k}}}$, normalizes the attention scores. The scaled attention scores are then used to weight the value vectors, resulting in an enhanced representation of local features. In addition to these steps, the mechanism integrates multiple convolutional filters inspired by the inception module [43] to capture local spatial information at various scales. A feature tensor *F* of shape *bxc* × *h* × *w* is initially reduced to *bxc* × *1* × *1* using a convolution of 1 × 1. Spatial local details are then extracted using dilated convolutions with kernel sizes of 3 × 3, 5 × 5, and 7 × 7, and dilations of 1, 2, and 3, respectively. These convolutions efficiently capture multiscale features. The results from these convolutions, along with the output from the 1 × 1 layer convolution, are merged into a tensor of size *bx4c* × *h* × *w*. A final 1 × 1 convolution then reduces this concatenated tensor to a *bx1* × *h* × *w* local spatial attention map, *Yls*. This attention map highlights and localizes important regions within the input data, enhancing the model’s ability to focus on significant areas. Similar to GLCA, the final Global–Local Spatial Attention (GLSA) output is obtained by ${F}_{s}^{l}=F \times Yls$ and $Fg = {F}_{s}^{l} \times Ygs + {F}_{s}^{l}$.

### Slice Interaction Module (SIM)

Both the GLCA and GLSA modules incorporate the slice interaction module (SIM), where the batch dimension is used to simulate depth, effectively allowing data processing in a pseudo-3D manner, as seen in Fig. 2f. That is, without the SIM, the network would be two-dimensional. In typical 3D processing, data is treated as a tensor with dimensions corresponding to width, height, and depth. However, in this pseudo-3D approach, the batch dimension is repurposed as the depth dimension. Each slice (or image) in the batch corresponds to a different object layer along the depth axis. Thus, a 2D image stack, where each image in the batch represents a different slice of the same object, effectively becomes a pseudo-3D volume. Queries, keys, and values are computed using linear transformations applied to the input tensor *x*. Let *x ∈ R*^*b*×*hw*×*c*^, where *b* is the batch size (depth), *hw* represents the spatial dimensions (height × width), and *c* is the number of channels. These projections are reshaped and permuted to facilitate multi-head attention, as explained in [44], where each head independently attends to different input parts. The core of the mechanism involves computing dot-product attention, which measures the relationships between different positions in the input tensor. This attention is scaled to ensure stability and then used to compute weighted averages of the values, capturing dependencies across both the spatial dimensions (height and width) and the pseudo-depth (batch dimension). The SIM captures cross-slice dependencies after combining global and local attention.

### Fusion

As depicted in Fig. 1, we integrated the local *Fl*, the global *Fg*, and the original feature maps *F*. Instead of using standard techniques like concatenation or addition to combining these features, a weighted average technique was adopted. The weights, such as *wlo*, *wgo*, and *wt*, were derived via softmax to three variable parameters, which produced the fused global–local attention map, *Fgl* = *wloFl* + *wgoFg* + *wtF*, which has dimensions *bxc* × *h* × *w*. The key distinction between proposed and existing methods lies in how attention maps are generated. The proposed method forms these maps by merging multiscale encoder features rather than treating each stage separately. This approach captures long-range dependencies across various encoder stages, unlike existing attentions, which operate within individual stages.

### Datasets

We trained and evaluated GLCSA on in-house prostate MRI (UAB) and ProstaeX datasets. The in-house UAB dataset includes 44 patients who underwent a prostate mpMRI scan due to either active surveillance or elevated PSA levels [41]. All subjects were imaged using a 3 T MRI scanner (SIEMENS Prisma) equipped with a dual-channel transmit RF coil. A turbo spin-echo (TSE) T2-weighted MRI sequence was used to delineate the boundaries of the prostate. Out of 44 patients, we used 34 patients’ data for training and the remaining 10 for testing. ProstateX is a publicly available dataset [42]. The ProstateX dataset comprises 204 mpMRIs, including high-resolution T2-weighted MRI (one scan per patient), acquired using two different Siemens 3 T MRI scanners (T MAGNETOM and Skyra). Of 204 images, 183 were used for training and 21 for testing.

### Dataset Pre-processing

All images were single-channeled (grayscale). The images from both datasets were originally *n* × 320 × 320, where *n* was the number of slices. We center-cropped the images as that is where the prostate typically lies. Center cropping is a technique used to extract a specific region from an image, focusing on its central portion. This method involves calculating the coordinates for the crop by determining the image’s dimensions (320 × 320) and subtracting half of the desired crop width and height from the image’s center. By doing so, a symmetrical crop is obtained, allowing for consistent dimensions while retaining the most relevant features of the image. The cropped images were then resized to 20 × 128 × 128 (*d* × *w* × *h*) and normalized from 0 to 255. A multiclass segmentation was implemented, where class 0 corresponds to the background and classes 1 and 2 correspond to TZ and PZ, respectively. Random flipping and rotation were implemented for data augmentation to double the size of the datasets.

### Implementation and Training

**Fig. 3:**
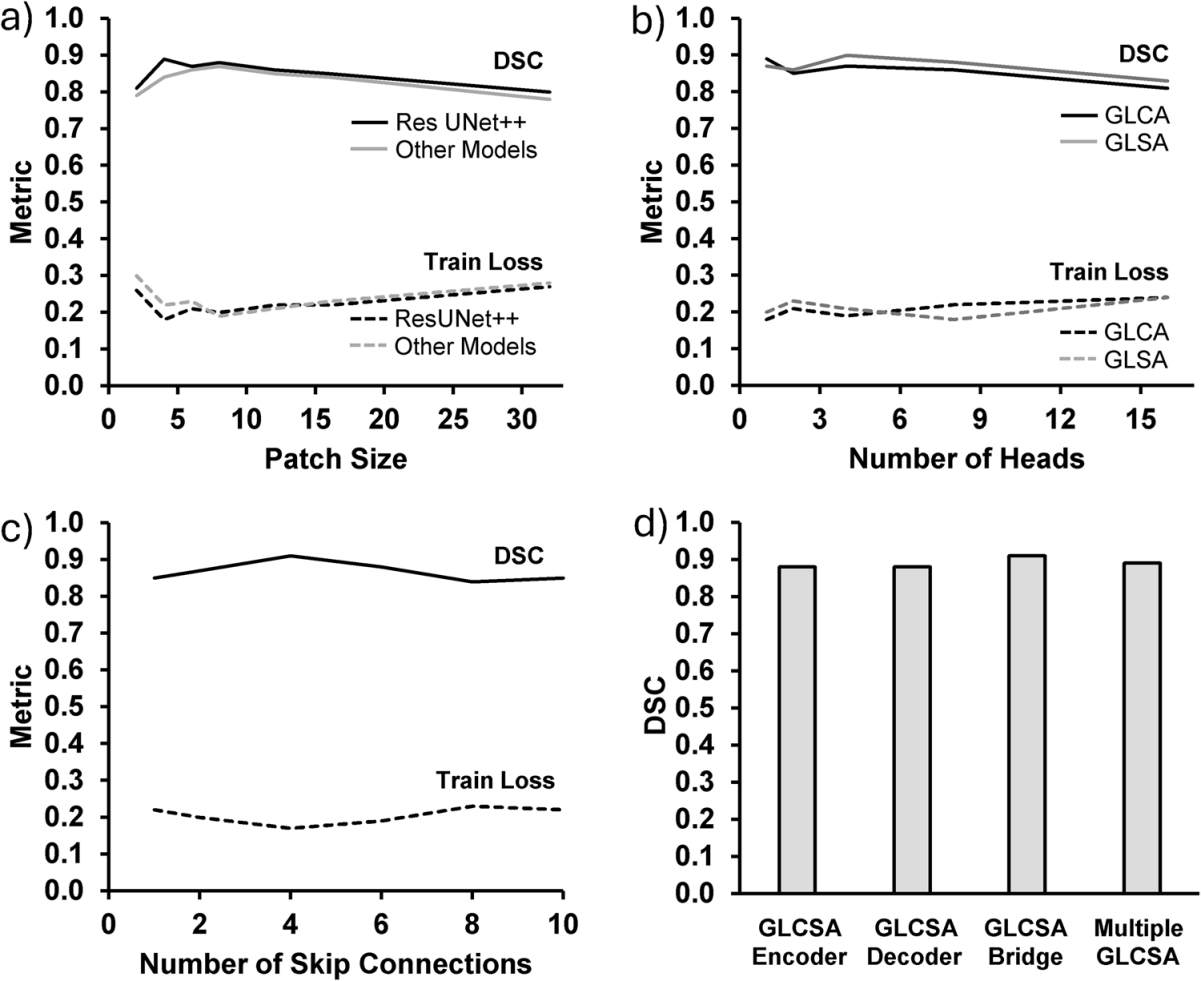
Model performance with varying parameters. Performance (Dice Similarity Coefficient (DSC)) and training loss with varying **a** patch size, **b** number of attention heads on GLCA and GLSA modules, and **c** number of skip connections. **d** DSCs when GLCSA is integrated into different network parts

For the inter-model evaluation with and without GLCSA, seven UNet models were used such as UNet [43], UNet +  + [44], VNet [45], Multi-ResUNet [46], R2UNet + [47], SEUNet [48], and Res UNet +  + [49]. For all the modes except the Multi-ResUNet, the GLCSA block was positioned between the decoder and encoder stages. In the Multi-ResUNet architecture, the GLCSA block was integrated to refine their output wherever the residual paths end. UNet +  + , composed of architectures, employed three distinct skip-connection strategies. First, the encoder (VGG19) was connected to its corresponding decoder. Second, the features from the initial encoder were connected to the subsequent decoder. Third, the second encoder was connected to its decoder. A GLCSA block was placed into each skip-connection pathway. After performing a grid search, patches 4 and 8 were set to ResUNet +  + and other models. The number of GLCA and GLSA heads was configured to 1 and 4, respectively. Figure 3 illustrates various parameter studies and their impact on the DSC and training loss. In Fig. 3a, DSC peaks at patch sizes 4 and 8, showing an optimal balance between feature extraction and efficiency, while larger patches dilute local information. Training loss decreases with smaller patches but stabilizes for larger sizes. Figure 3b shows that DSC is highest with 1 GLCA and 4 GLSA heads, indicating that these configurations effectively balance context aggregation without introducing redundancy, as excessive heads increase training loss. Figure 3c reveals that four skip connections yield the best performance, balancing spatial and semantic information flow, whereas fewer or excessive connections reduce effectiveness. Lastly, Fig. 3d shows GLCSA as a bridge (between encoder and decoder) that achieves the highest DSC by effectively capturing global context and balancing encoder-decoder flow, with multiple placements offering improvements but at a higher computational cost. The Adam optimizer with a starting learning rate of 0.0001 was employed across all models [50]. The training loss function was the multiclass Dice Loss [51]. Training performance metrics were set to the multiclassice Similarity Coefficient (DSC) and Intersection over Union (IoU) scores. All models were trained for 200 epochs. For the comparative analysis, we compared GLCSA-UNet with other popular 2D networks such as r2UNet [52] and AttnR2UNet [53], 2.5D networks such as CAT [27] and CSAM [54], and 3D networks such as 3D VNet [55] and 3D UNet [56]. The training and testing protocols were the same for all models.

## Results

Tables 1 and 2 summarize inter-model comparisons for the UAB and ProstateX datasets, respectively. Each model was evaluated both with and without the integration of GLCSA. On average, the number of parameters increased by 1.27% following the incorporation of GLCSA. For the UAB dataset, the mean test DSC in the TZ and PZ improved by 0.74% and 11.75%, respectively. Similarly, for the ProstateX dataset, GLCSA integration resulted in average DSC increases of 7.34% in the TZ and 24.80% in the PZ. A similar increase was noticed with the MSD values as well. For the UAB dataset, the mean test MSD in the TZ and PZ improved by 7.95% and 16.23%, respectively. Similarly, for the ProstateX dataset, GLCSA integration resulted in average increases of 11.85% in the TZ and 11.91% in the PZ on MSD.

**Table 1:** Performance metrics of seven conventional models with or without GLCSA on the UAB dataset. Average Dice Similarity Coefficient (DSC), worst-case DSC, and average Mean Surface Distance (MSD) of conventional models with and without the use of proposed GLCSA for segmenting the peripheral (PZ) and transition (TZ) prostate zones on the unseen UAB test set. The best model performance for TZ or PZ segmentation is highlighted in bold when assessed by the average test DSC or MSD

Model	Region	Test average DSC ↑		Worst-case DSC		Test average MSD	
		With GLCSA	Without GLCSA	With GLCSA	Without GLCSA	With GLCSA	Without GLCSA
UNet	TZ	**0.88 ± 0.04**	0.84 ± 0.03	0.80	0.76	**0.95 ± 0.36**	1.62 ± 0.34
	PZ	**0.68 ± 0.10**	0.60 ± 0.10	0.50	0.45	1.18 ± 0.36	1.65 ± 0.40
UNet + +	TZ	0.88 ± 0.04	0.91 ± 0.02	0.80	0.88	0.94 ± 0.27	0.86 ± 0.21
	PZ	0.68 ± 0.11	0.73 ± 0.08	0.45	0.63	1.22 ± 0.47	1.22 ± 0.28
MultiRes UNet	TZ	0.89 ± 0.04	0.89 ± 0.03	0.82	0.84	**0.87 ± 0.26**	1.10 ± 0.35
	PZ	**0.72 ± 0.08**	0.66 ± 0.11	0.60	0.52	**0.98 ± 0.23**	1.07 ± 0.24
Res UNet + +	TZ	0.87 ± 0.03	0.86 ± 0.05	0.79	0.76	**0.96 ± 0.25**	1.08 ± 0.23
	PZ	**0.68 ± 0.09**	0.60 ± 0.08	0.56	0.42	0.86 ± 0.24	1.30 ± 0.24
VNet	TZ	**0.87 ± 0.04**	0.82 ± 0.09	0.79	0.56	1.10 ± 0.26	1.65 ± 0.92
	PZ	**0.64 ± 0.08**	0.55 ± 0.15	0.50	0.29	**1.45 ± 0.34**	2.90 ± 1.38
SEUNet	TZ	0.85 ± 0.08	0.85 ± 0.07	0.62	0.66	**1.04 ± 0.35**	1.12 ± 0.34
	PZ	**0.65 ± 0.10**	0.59 ± 0.08	0.44	0.470	1.56 ± 0.45	1.36 ± 0.35
r2 UNet +	TZ	0.82 ± 0.09	0.85 ± 0.07	0.57	0.68	1.64 ± 0.86	1.44 ± 0.70
	PZ	**0.52 ± 0.14**	0.41 ± 0.11	0.29	0.22	**2.74 ± 0.97**	6.50 ± 0.96

**Table 2:** Performance metrics of seven conventional models with or without GLCSA on the ProstateX dataset. Average Dice Similarity Coefficient (DSC), worst-case DSC, and average Mean Surface Distance (MSD) of conventional models with and without the use of proposed GLCSA for segmenting the peripheral (PZ) and transition (TZ) prostate zones on the unseen ProstateX test set. The best model performance for TZ or PZ segmentation is highlighted in bold when assessed by the average DSC or MSD

Model	Region	Test average DSC		Worst-case DSC		Test average MSD	
		With GLCSA	Without GLCSA	With GLCSA	Without GLCSA	With GLCSA	Without GLCSA
UNet	TZ	**0.80 ± 0.09**	0.77 ± 0.09	0.46	0.43	**1.48 ± 0.38**	1.76 ± 0.45
	PZ	**0.76 ± 0.06**	0.72 ± 0.08	0.66	0.53	**0.98 ± 0.49**	1.20 ± 0.48
UNet + +	TZ	0.77 ± 0.11	0.78 ± 0.08	0.37	0.48	**1.57 ± 0.28**	1.64 ± 0.39
	PZ	0.74 ± 0.07	0.75 ± 0.06	0.61	0.65	**1.10 ± 0.50**	0.99 ± 0.30
MultiRes UNet	TZ	**0.78 ± 0.10**	0.77 ± 0.11	0.39	0.34	1.59 ± 0.34	1.53 ± 0.42
	PZ	**0.75 ± 0.07**	0.68 ± 0.08	0.62	0.46	**0.97 ± 0.40**	1.19 ± 0.31
Res UNet + +	TZ	**0.75 ± 0.11**	0.34 ± 0.08	0.37	0.16	**1.62 ± 0.35**	2.62 ± 0.53
	PZ	**0.70 ± 0.09**	0.57 ± 0.12	0.50	0.33	**1.11 ± 0.51**	1.94 ± 1.06
VNet	TZ	**0.73 ± 0.11**	0.70 ± 0.08	0.33	0.40	**1.89 ± 0.28**	2.07 ± 0.27
	PZ	**0.70 ± 0.08**	0.68 ± 0.10	0.55	0.49	**1.32 ± 0.58**	1.48 ± 0.73
SEUNet	TZ	0.73 ± 0.10	0.77 ± 0.09	0.41	0.43	1.80 ± 0.47	1.66 ± 0.40
	PZ	0.72 ± 0.10	0.74 ± 0.07	0.43	0.61	1.29 ± 0.84	1.11 ± 0.56
r2 UNet +	TZ	**0.69 ± 0.10**	0.46 ± 0.07	0.35	0.33	**1.98 ± 0.47**	2.76 ± 0.67
	PZ	**0.66 ± 0.10**	0.58 ± 0.12	0.43	0.36	**1.77 ± 0.89**	2.22 ± 1.09

Additionally, the tables reveal notable variations in standard deviations (SD) across TZ and PZ for both DSC and MSD metrics, as well as between cases with and without GLCSA. For DSC, the SD is generally smaller for TZ compared to PZ, indicating more consistent segmentation in TZ, which may be due to its more straightforward structure or more explicit boundaries. Including GLCSA generally reduces the SD for DSC, reflecting improved consistency in segmentation across models. For MSD, the SD is higher in PZ than TZ, likely due to increased anatomical complexity and segmentation challenges. The absence of GLCSA often results in higher SD for MSD, especially in PZ, further indicating that GLCSA contributes to reducing variability and improving model reliability. This suggests that including GLCSA enhances segmentation robustness, particularly in the more complex PZ region. Additionally, for the UAB dataset, the Wilcoxon signed-rank test revealed a statistically significant improvement with GLCSA, with a *p* value of 0.023 for DSC and 0.019 for MSD (Mean Surface Distance), both significant at the *α* = 0.05 level. Similarly, for the ProstateX dataset, the test confirmed significance, with a *p* value of 0.030 for DSC and 0.013 for MSD, demonstrating that GLCSA consistently enhances segmentation performance across these datasets.

**Fig. 4:**
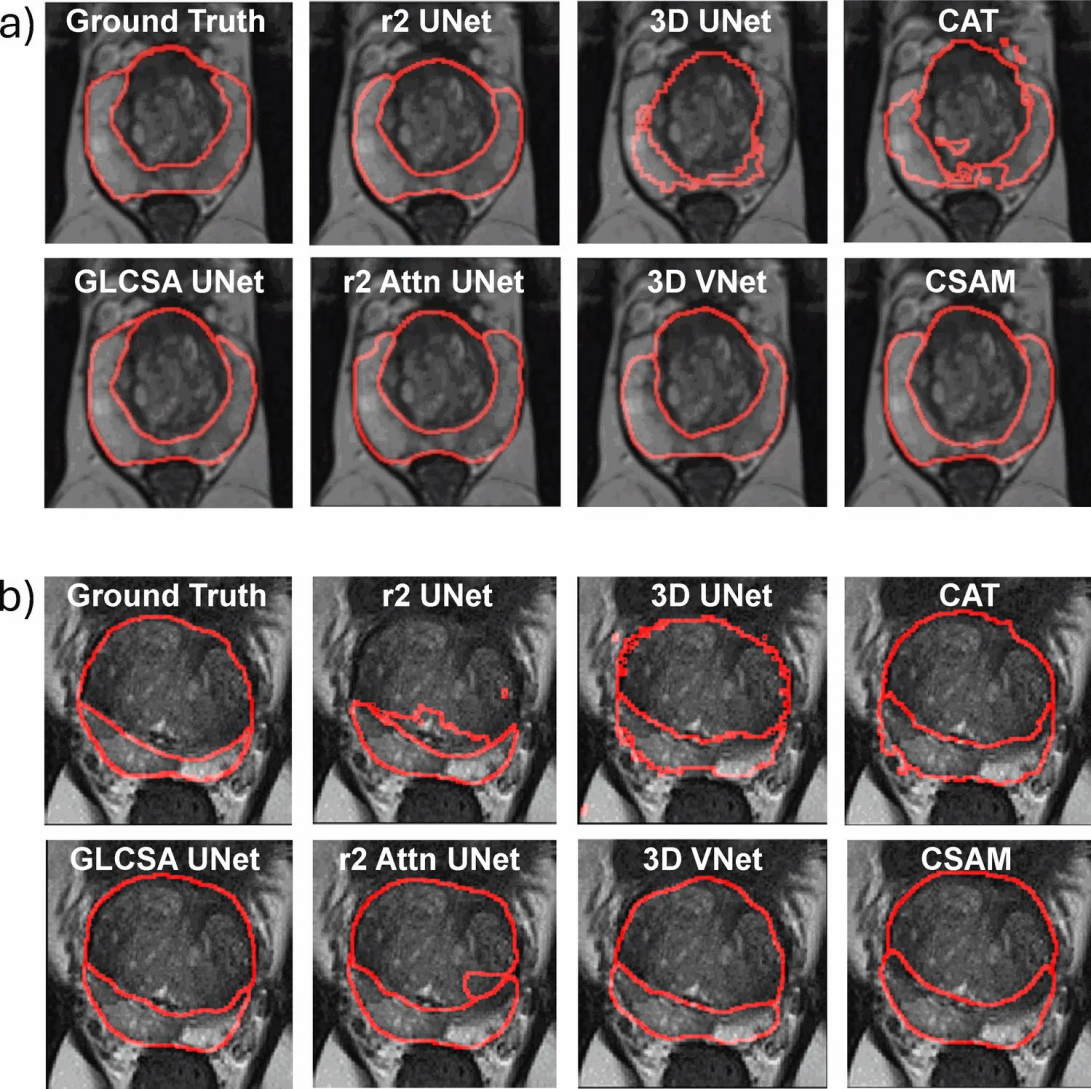
Representative prostate images with peripheral and transition zones determined by GLCSA UNet and six other 2D, 2.5D, or 3D models. **a**, **b** Representative prostate images with the boundaries of peripheral and transition zones determined manually (ground truth) or by GLCSA UNet, r2 UNet, 3D UNet, CAT, r2 Attn UNet, 3D Vnet, and CSAM, when **a** UAB or **b** ProstateX test datasets were employed

Table 3 compares the GLCSA UNet against six different 2D, 2.5D, and 3D models. Figure 4 presents representative image segmentations with these models from the unseen UAB or ProstateX test sets. By reducing misclassifications, the GLCSA method demonstrates superior capability in distinguishing distinct regions, such as the PZ and TZ. Additionally, GLCSA enhances the preservation of shape integrity and ensures more consistent boundary delineation. This combination improves the reliability of segmentation tasks and facilitates the accurate identification of anatomical structures.

**Table 3:** GLCSA UNet performance metrics compared with six other 2D, 2.5D, and 3D models. Average Dice Similarity Coefficient (DSC) and average Mean Square Distance (MSD) of the proposed model (GLCSA UNet) in comparison with popular 2D, 2.5D, and 3D networks for segmenting the peripheral (PZ) and transition (TZ) prostate zones on the unseen UAB or ProstateX test set. The best model performance for TZ or PZ segmentation in the UAB or ProstateX dataset is highlighted in bold when assessed by the average DSC or MSD

Network	Model	Region	Test average DSC		Test average MSD	
			UAB	ProstateX	UAB	ProstateX
2D	r2 UNet	TZ	0.80 ± 0.11	0.73 ± 0.09	1.73 ± 0.87	**1.08 ± 0.79**
		PZ	0.62 ± 0.12	0.67 ± 0.15	2.94 ± 1.73	2.31 ± 1.55
	AttnR2UNet	TZ	0.83 ± 0.06	0.77 ± 0.10	1.90 ± 0.87	1.49 ± 0.33
		PZ	0.51 ± 0.16	0.76 ± 0.07	2.63 ± 1.02	1.02 ± 0.43
2.5D	CAT	TZ	0.84 ± 0.04	0.77 ± 0.11	1.20 ± 0.52	1.68 ± 0.34
		PZ	0.59 ± 0.13	0.69 ± 0.08	1.47 ± 0.36	1.29 ± 0.41
	CSAM	TZ	0.87 ± 0.04	0.79 ± 0.09	1.08 ± 0.41	1.61 ± 0.49
		PZ	0.61 ± 0.08	0.75 ± 0.06	1.47 ± 0.29	0.98 ± 0.30
3D	3D VNet	TZ	0.88 ± 0.83	0.81 ± 0.10	0.74 ± 0.13	1.51 ± 0.46
		PZ	0.62 ± 0.10	0.75 ± 0.06	2.92 ± 1.93	0.96 ± 0.33
	3D UNet	TZ	0.82 ± 0.04	0.79 ± 0.10	1.22 ± 0.36	1.58 ± 0.38
		PZ	0.37 ± 0.13	0.70 ± 0.72	4.79 ± 2.20	1.48 ± 0.58
2.5D	GLCSA UNet (proposed)	TZ	0.85 ± 0.08	0.80 ± 0.09	1.14 ± 0.51	1.48 ± 0.38
		PZ	0.65 ± 0.11	0.76 ± 0.06	1.21 ± 0.33	0.98 ± 0.49

In the 2D category, both r2 UNet and AttnR2UNet demonstrated solid performance across the UAB and ProstateX datasets. For the UAB dataset, r2 UNet achieved DSC values of 0.80 for TZ and 0.62 for PZ, with corresponding MSD values of 1.73 and 2.94, respectively. In the ProstateX dataset, its DSC values dropped to 0.73 for TZ and 0.67 for PZ, with MSD values of 1.08 and 2.31. AttnR2UNet showed superior performance in the TZ, with DSC values of 0.83 and 0.77 and MSD values of 1.90 and 1.49 in the UAB and ProstateX datasets, respectively. However, it struggled in the PZ, where its DSC dropped from 0.76 (ProstateX) to 0.51 (UAB), with MSD values of 1.02 and 2.63.

In the 2.5D category, CAT and CSAM models produced competitive results. CSAM stood out with the highest DSC for TZ at 0.87 and 0.79 and MSD values of 1.08 and 1.61 in the UAB and ProstateX datasets, respectively. CAT followed closely, with DSC values of 0.84 and 0.77 and MSD values of 1.20 and 1.68 for TZ in the UAB and ProstateX datasets, respectively. Both CAT and CSAM also performed well in the PZ, with CSAM achieving better DSC (0.61 UAB, 0.75 ProstateX) and MSD (1.47 UAB, 0.98 ProstateX). The proposed GLCSA UNet demonstrated robust performance, with DSC values for TZ and PZ of 0.85 and 0.65 (UAB) and 0.80 and 0.76 (ProstateX), accompanied by MSD values of 1.14 and 1.21 (UAB) and 1.48 and 0.98 (ProstateX).

In the 3D category, 3D VNet led with the highest DSC for TZ in both datasets (0.88 UAB, 0.81 ProstateX) and the lowest MSD values (0.74 UAB, 1.51 ProstateX). Conversely, 3D UNet exhibited more variability, particularly in the PZ, with DSC values of 0.37 (UAB) and 0.70 (ProstateX) and MSD values of 4.79 and 1.48. Overall, the GLCSA UNet displayed high performance across both datasets, with notable improvements in PZ segmentation compared to other models in the 2.5D category, demonstrating its effectiveness across different datasets and segmentation tasks. Table 4 summarizes the results of the ablation study conducted on the test dataset for the targeted regions. Initially, we evaluated the performance with only one component—GLSA or GLCA—and observed that the model outperformed the baseline UNet without GLCSA in both cases. However, the DSC values for the individual components were lower than when combined, indicating that the GLCA plays a crucial role even in grayscale images. Furthermore, no significant difference in DSC was observed between applying GLCA followed by GLSA versus GLSA followed by GLCA, suggesting that the order of attention application does not substantially impact performance. The current approach employed the GLCA-GLSA sequence, showing that all attention types effectively complement each other, irrespective of the order in which they are applied.

**Table 4:** Ablation Study on UAB dataset. Dice Similarity Coefficient (DSC) on the transition zone of the UAB test set on various ablated conditions (*DSC* Dice Similarity Coefficient, *GLSA* Global–Local Spatial, *GLCA* Global–Local Channel, *NSC* Nested Skip, *FSC* Full-Scale Skip, *CSC* Concatenated Skip, *Seq* sequential, *LA* local attention, *GL* global)

Region	Attention		Local attention		Embedding		Skip connections			Fusion technique			DSC
	Only GLSA	Only GLCA	LA 1	LA 2	Patch	GL	NSC	FSC	CSC	Seq	Sum	Parallel	
TZ	✔		✔		✔			✔		✔			0.87
		✔	✔		✔			✔		✔			0.85
	✔	✔	✔		✔			✔		✔			0.88
	✔	✔		✔	✔			✔		✔			0.86
	✔	✔	✔		✔			✔		✔			0.87
	✔	✔	✔			✔		✔		✔			0.88
	✔	✔	✔		✔		✔			✔			0.87
	✔	✔	✔		✔			✔		✔			0.86
	✔	✔	✔		✔				✔	✔			0.86
PZ	✔	✔	✔		✔			✔		✔			0.68
	✔	✔	✔		✔			✔			✔		0.67
	✔	✔	✔		✔			✔				✔	0.66

## Discussion

The proposed GLCSA UNet achieves a practical improvement in segmentation performance while maintaining a lower parameter count (47 M) compared to other 2.5D and 3D models like CSAM (61 M) and 3D VNet (57 M). This highlights its efficiency in delivering high accuracy with reduced computational complexity. We comprehensively evaluated several fusion techniques to integrate attention mechanism modules, explicitly focusing on the Global–Local Channel Attention (GLCA) and Global–Local Spatial Attention (GLSA) components. The fusion techniques examined included sequential fusion (applying GLCA and GLSA in sequence), summation fusion (summing the outputs of GLCA and GLSA), and parallel fusion (executing GLSA and GLCA in parallel and summing the outputs) [57]. Our experiments revealed that although parallel and summation fusions led to slight accuracy improvements, the sequential method consistently delivered the best performance across multiple metrics. We hypothesize that this superior performance stems from the sequential method’s ability to more effectively capture hierarchical features, enabling each attention mechanism to build on the information extracted by the preceding one.

We conducted additional experiments with varying sequence orders (GLCA followed by GLSA, and vice versa) and found that the order of application also impacts the model’s performance. Specifically, applying GLCA followed by GLSA resulted in a 2.3% improvement in the Dice score compared to the reverse order. This finding suggests that the interaction between different attention mechanisms can have complex effects on model performance, highlighting the importance of careful configuration and extensive experimentation. In our current model, we implemented a local attention mechanism. To explore potential improvements, we also tested a modified approach. Our current approach (LA1) uses a standard local attention implementation, while the modified approach (LA2) sets the batch size to 1 and treats each 2D slice as having a pseudo-depth of 1. The LA2 approach showed promising results when used with GLSA alone, improving the Dice score by approximately 3.5%. However, when both GLCA and GLSA were combined, we observed a performance decrease of about 2.1% compared to the LA1 approach.

We conducted an ablation study, isolating the effects of GLCA and GLSA in both LA1 and LA2 configurations. Results indicated that while LA2 enhances spatial attention capabilities (GLSA), it potentially interferes with the channel attention mechanism (GLCA) when combined. These findings underscore the complex interplay between different attention mechanisms and their implementation details, emphasizing the need for thorough experimentation to optimize model architecture.

We explored two distinct embedding techniques to process the input images: patch embedding and global embedding. Patch embedding divides the input image into fixed-size patches using a pooling operation, projecting it into a sequence format. This method increases the number of parameters (~ 153 K) due to the use of kernels. In contrast, global embedding resizes the entire image using interpolation, preserving more global context without reducing it into patches. Our experiments revealed that global embedding slightly outperformed patch embedding in terms of the Dice Similarity Coefficient (DSC), with an improvement of approximately 1.8%. This suggests that preserving the overall image context benefits our specific task.

However, it is worth noting that patch embedding introduces a larger number of parameters due to the use of kernels. We conducted additional experiments with varying patch sizes and kernel configurations to investigate whether this increased parameter count could be leveraged for improved performance. Results showed that smaller patch sizes (e.g., 4 × 4) improved local feature capture and significantly increased computational complexity. Conversely, larger patch sizes (e.g., 16 × 16) reduced computational load but at the cost of fine-grained feature detection. These findings underscore the trade-off between global context preservation and local feature capture, suggesting that an optimal balance depends on the specific requirements of the task at hand.

All UNet variants discussed in this paper utilize nested skip connections (NSCs) [44]. We replaced NSCs with full-scale connections (FSCs) [58] and Concatenated Skip Connections (CSCs) [59] for comparison. Table 4 shows that models with NSCs performed slightly better than those with CSCs and FSCs, suggesting that GLCSA functions more effectively with dense skip connections. Specifically, we observed that NSCs outperformed FSCs by a margin of 1.5% in terms of DSC and showed a 0.8% improvement over CSCs in overall accuracy. These results suggest that GLCSA functions more effectively with dense skip connections provided by NSCs. To explore this phenomenon further, we performed an in-depth analysis of feature map activations at various stages of the network. Our findings indicated that NSCs promote better gradient flow and feature reuse across different layers of the network hierarchy. This enhanced information flow synergizes particularly well with the global–local attention mechanisms in GLCSA, enabling more effective integration of multiscale features.

The sequential fusion of GLCA and GLSA, combined with global embedding and nested skip connections, has proven to be the most effective approach in our study. Although this research offers valuable insights into attention mechanisms for medical image segmentation, the small sample size and limited dataset diversity may have led to a bias toward dataset-specific features. To overcome these challenges, future work should prioritize using larger, more diverse datasets, enhance 3D modeling, and improve interpretability for practical clinical applications.

Our future efforts will focus on advancing attention mechanisms, exploring deformable and axial attention, and investigating the potential of transformer-based architectures for medical image segmentation. We also aim to develop adaptive fusion techniques that dynamically adjust based on input characteristics and incorporate uncertainty estimation to provide confidence measures for segmentation outcomes. By refining these components, we seek to push the limits of segmentation performance and build more robust, accurate models for medical image analysis.

## Conclusions

We introduced a pseudo-3D GLCSA mechanism designed to enhance prostate zonal segmentation while maintaining computational efficiency. This multi-faceted attention mechanism increases segmentation accuracy by improving the model’s ability to capture complex features across multiple dimensions. By effectively merging multiscale, low-level encoder features, the model can extract fine-grained representations, helping to connect the pixel gap between low- and high-level features and enhancing segmentation precision. The method achieves computational efficiency by employing 2D convolutions while functioning similarly to 3D, reducing the computational burden. Future work will explore the performance of GLCSA and its 2D and 3D variants across different datasets.

## Data Availability

We employed our own institutional dataset and one of the publicly available datasets which is cited.

## Code Availability

The proposed GLCSA model’s code is available in the following GitHub Repository: https://github.com/chetana348/GLCSA-for-Prostate-Zonal-Segmentation.
